# Level of Adherence to the Mediterranean Diet and Weight Status among Adolescent Female Gymnasts: A Cross-Sectional Study

**DOI:** 10.3390/children8121135

**Published:** 2021-12-04

**Authors:** Ioanna Kontele, Maria G. Grammatikopoulou, Tonia Vassilakou

**Affiliations:** 1 Department of Public Health Policy, School of Public Health, University of West Attica, 196 Alexandras Avenue, GR-11521 Athens, Greece; 2 Department of Nutritional Sciences & Dietetics, Faculty of Health Sciences, International Hellenic University, Alexander Campus, GR-57001 Thessaloniki, Greece; maria@ihu.gr

**Keywords:** adolescence, Mediterranean Diet, sports, adolescent athlete, gymnastics, low energy availability, relative energy deficiency in sport syndrome (RED-S), underweight, diet quality, nuts, healthy diet, competition

## Abstract

Adolescent female gymnasts are a vulnerable population in terms of their diet, as their nutritional needs are higher due to their growth and high daily training demands. The Mediterranean diet (MD) is a well-known dietary pattern that is associated with a greater nutritional adequacy and a lower prevalence of overweight. The aim of this cross-sectional study was to evaluate the degree of adherence to the MD among adolescent female athletes who participated in all disciplines of gymnastics in Greece, as well as to explore the potential correlations between MD adherence, body weight, and body mass index (BMI). A total of 269 female gymnasts (between 11–18 years old) completed the Mediterranean Diet Quality Index (KIDMED) and reported their weight and height. Approximately 10% of the participants were underweight and a mere 5.6% were classified as overweight. A high adherence to the MD was reported by 34.9% of the sample and this was correlated with a healthier BMI. Moreover, specific eating habits, such as eating nuts 2–3 times per week and eating fast-food less than once-weekly, were also associated with BMI. Athletes, parents, and coaches of gymnastics should be informed and educated regarding balanced nutrition habits. Future studies could evaluate adherence to MD, as well as the factors that influence the eating behaviors of adolescent female and male athletes in various sports.

## 1. Introduction

Adequate nutrition, both in terms of quantity and quality, is especially important during adolescence in order to meet the increased demands of development and promote optimal physical, cognitive, and psychological health [1]. Although nutritional deficits in adolescence are not as severe as they appear in childhood and infancy, adolescents are considered as a nutritionally vulnerable population for a variety of reasons, including their greater developmental requirements, their usually unhealthy eating habits, and their lifestyle particularities [2,3,4]. At the same time, adolescence consists of a critical period for the establishment of healthy eating habits, that will be kept during the rest of life [5]. According to studies primarily from Western countries, adolescents’ diets appear to be less adequate than those of adults [6]. Adolescents’ unhealthy eating habits include frequent snacking with high-energy and low nutrient density foods, skipping meals, consuming low amounts of fruits and vegetables, and eating fast food frequently [6,7,8,9]. On the other hand, many adolescents, particularly females, drastically limit their energy intake in their attempt to lose body weight [10,11].

Attaining a healthy and balanced nutrition is particularly important for adolescent athletes, as they experience several anatomical and physiological changes, heightening their nutritional requirements for optimal growth, while in parallel, having to cater for increased energy expenditure as a result of a hectic, daily training schedule [12,13,14,15]. An inability to meet these requirements may lead to the development of relative energy deficiency in sport (RED-S) syndrome [12]. At the same time, adverse effects on reproductive and bone health may occur due to low energy availability, leading to the development of the female athlete triad [16]. As adolescence is the period of peak bone mass accumulation, the triad can result in very serious bone health problems in later life [13].

Female gymnasts in particular are a nutritionally vulnerable population, with high nutritional demands due to the long hours of daily training [17]. They may compete at the international level from their early adolescence, often training up to six days a week for 4–6 h on a daily basis [18]. At the same time, gymnasts train in a sport where a low body fat level is considered to be a “benefit”. Reduced body mass, especially fat mass, combined with maximum muscle strength enhances flexibility, strength, and exercise technique, as well as increases power-to-weight ratio. This is important for optimum performance in gymnastics, where athletes move their body mass rapidly through space, overcoming gravitational resistance [18,19,20]. Respectively, excessive fat mass is a disadvantage, as it decreases the efficiency of movements [21]. Previous research has shown that small bodies and a lower body mass are characteristics demonstrated by elite gymnasts, and the measurements of the most successful elite female gymnasts suggest low body fat composition [19]. Lower BMI has also been related to better performance in a study of elite female gymnasts, although this relationship was curvilinear. There was a trend toward thinner athletes performing better, but the effects of thinness reached a point where performance was more negative as BMI became very low, indicating that a low weight, but one within normal limits, is more beneficial [22]. Another important issue regarding weight in gymnastics is that a lean body shape is preferable for aesthetic reasons. Although points for weight or physique are not allocated in gymnastics, it is believed that the athlete physique may influence judges’ scores for artistic impression [23,24]. Therefore, athletes of gymnastics are often characterized by a lean shape, and even mild and moderate thinness occur in world class gymnasts. Alongside this, maturity status is a factor that affects weight-for-height relationships, and later maturation is often reported [18,25].

Ιn order to attain the ideal physical standards for their sport, female gymnasts often engage in low-calorie diets and inappropriate dietary strategies in their attempt to regulate their body weight and/or body shape [26,27,28,29,30]. Several studies have shown that adolescent gymnasts are in a low energy availability status, being at a high risk for the development of RED-S and the female athlete triad, as a result of low energy diets and unhealthy methods to control their weight [30,31,32,33,34,35]. In parallel, research also indicates that adolescent gymnasts are at greater risk of exhibiting disordered eating behaviors and a plethora of micronutrient deficiencies and inadequate intakes, involving mainly calcium, iron, folic acid, vitamin D, and zinc [30,31,32,36,37].

However, evaluating a population’s diet quality through usual intake records consists of a difficult process, while it is also known that foods and nutrients act synergistically, influencing health status. To correct for this, it has been suggested that overall dietary patterns should be studied, instead of single nutrient intakes [38,39,40,41]. The Mediterranean diet (MD) is a well-known dietary pattern characterized by the high consumption of vegetables, fruit, fish, pulses, and grains, a moderate consumption of milk and dairy products, a low intake of meat and by-products, and a high monounsaturated (MUFA)/saturated fat (SFA) ratio [42,43]. High adherence to the MD has been associated with a reduced risk of cardiovascular disease (CVDs), several types of cancer, diabetes, and overall mortality [44,45,46,47]. These benefits have not only been attributed to the components and specific food-groups that are consumed, but also to the cooking techniques employed, as well as eating and lifestyle behaviors [42,44]. Adherence to the MD has also been associated with improved health outcomes in children and adolescents, especially with regard to their weight status, with a greater adherence being associated with a lower prevalence of overweight and obesity [8,48,49,50,51]. A limited number of studies have investigated the association between MD adherence and the anthropometric characteristics of adolescent athletes, and their results are ambiguous [52,53].

Moreover, research on female gymnasts in particular, who consist of a nutritionally vulnerable group, is extremely limited. To address this gap, the aim of the present study was to evaluate diet quality via adherence to the MD in relation to body weight status, in a sample of adolescent female gymnasts.

## 2. Materials and Methods

### 2.1. Participant Recruitment

A total of 319 Greek female gymnasts aged between 11–18 years old participated in the study. The athletes were training in one of the seven disciplines of gymnastics currently available in Greece (artistic and rhythmic gymnastics, trampoline, tumbling, acrobatics, aerobics, gymnastics for all), and they were training either in the national training centers situated in Athens and Thessaloniki, or in regional gymnastics clubs in Athens, Thessaloniki, Kavala, or Crete.

Data collection took place between August and November 2020. National coaches and local gymnastics clubs across the country were initially informed about the research protocol via email, as well as through the national federation’s website.

### 2.2. Inclusion and Exclusion Criteria

Among the clubs that expressed a desire to participate in the research project, the inclusion criteria for participation in the study involved (1) female athletes, (2) aged 11–18 years old, (3) training in one of the seven disciplines of gymnastics, (4) provided consent (personal and parental/guardian) for participation, (5) were able to understand the aim of the study and complete the questionnaires. Exclusion criteria involved (1) male athletes, (2) athletes aged <11, or >18 years of age, (3) athletes unable to provide information in the Greek language or understand the study’s protocol, (4) athletes with serious diagnoses related to nutrition (such as diabetes, celiac disease, anorexia nervosa, etc.), and (5) those who did not provide consent.

### 2.3. Ethical Permission, Consent and Anonymity

This cross-sectional study was a part of the project “Eating habits, weight pressures and disordered eating attitudes of adolescent female athletes of gymnastics in Greece”. The protocol was approved by the Ethics Committee of the University of West Attica (52760/21 July 2020) and by the Hellenic Gymnastics Federation (3151/28 August 2020). The study is reported based on the STROBE guidelines.

For the participation of athletes in the research project, an informed consent from a parent or guardian was requested. Parents were informed about the purpose of the study, the confidentiality and anonymity of the athletes, as well as the possibility of withdrawing at any time. The consent form provided the contact information of the lead researcher for any further questions. Parents were also asked to report if their child was suspected of having a nutrition-related disease (such as diabetes, celiac disease, or anorexia nervosa). In that case, the athlete was not eligible to participate. Then, with the permission of the coaches, researchers visited the training facilities and distributed a questionnaire only to those athletes whose parents had signed the consent form. Participants responded to the questionnaire anonymously and a researcher was available to answer questions and collect the completed questionnaires. Written information regarding the purpose of the study and clarifying the anonymity of the participants and the possibility of withdrawing were distributed along with the questionnaires. 

### 2.4. Questionnaires and Tools Applied

The first section of the questionnaire included questions regarding the performance/competition level, the years of training experience, the duration of training sessions, the competition frequency, and the perceived level of performance.

#### 2.4.1. Adherence to the MD

The level of adherence to the MD was evaluated through the Mediterranean Diet Quality Index (KIDMED), that was developed especially for young people (2–24 years old) [54]. The index consists of 16 distinct questions evaluating the frequency of consumption of food groups typical or atypical of the MD, including fruits, vegetables, fish, pulses, cereals, nuts, olive oil, yogurt, cheese, fast-food, and sweets, while individual questions also involve the possible omission of breakfast and its quality. Each item may receive a yes/no answer. Positive answers to questions related to negative habits receive a score of −1, whereas those related to positive habits associated with the MD are scored with one positive point (+1). The overall score ranges between 0 to 12. A score ≥8 is considered as indicative of an optimal MD adherence, a score between 4–7 indicates a MD of moderate quality, whereas a score ≤3 points to a low-quality diet. Greater scores are indicative of a greater adherence to the MD, and have been associated with greater nutritional adequacy, especially in terms of vitamins and minerals [54]. Apart from the total KIDMED, individual KIDMED components were also analyzed independently.

The score has been translated in the Greek language and used in samples of Greek children/adolescents in a plethora of research items [8,55,56].

#### 2.4.2. Social Desirability Scale

The social desirability scale (SDS) [57] is a 13-item tool investigating how social desirability influences the responses to self-reported questionnaires. A true or false answer scale is used for each item. A total of 8 items are scored with either 0 points for true notions, 1 point for false statements, and the remaining 5 items are scored with either 1 point for true, or 0 points for false statements. The total score ranges between 0 to 13, with a score > 9 being indicative of responses that are more likely to represent what is assumed to be desired, rather than the truth. For the purpose of our study, we applied the Greek version of the scale that has been translated and validated by Psychountaki et al. [58].

#### 2.4.3. Anthropometric Indices

The body weight and stature of each participant was self-reported. Body mass index (BMI) was calculated for each participant as body weight (kg), divided by height (m^2^). The World Obesity BMI cut-offs [59,60] were applied for the classification of weight status as underweight, normoweight, or overweight.

### 2.5. Statistical Analyses

Analyses were performed using SPSS version 22.0 (SPSS Inc., Chicago, IL, USA). For all analyses, the level of statistical significance was set at 0.05. Data were examined for normality, with no violations being determined. Continuous variables are presented as mean ± standard deviation and categorical variables are presented as frequencies and percentages.

The Pearson’s r correlation coefficient was used for investigating the relationships between continuous variables, while for the categorical variables a Chi-squared test was performed with the Fisher’s exact, when deemed necessary. Except for the total KIDMED score, which was used to classify adherence to the MD, individual items of the tool were also analyzed (e.g., skipping breakfast, eating fast-food etc.). Independent samples *t*-tests were used to examine the differences in BMI between the groups, according to the level of adherence to the MD, or affirmative/negative answers in individual KIDMED items. A one-way ANOVA with post-hoc analyses was performed to examine the differences in anthropometric characteristics between the groups of athletes of the different disciplines, and between the levels of adherence to the MD.

Finally, a linear regression model was used to identify the ability of the KIDMED score to predict BMI. Moreover, multiple regression analysis with the enter method was performed to predict BMI, using individual items of the KIDMED as independent variables.

For the purpose of the analyses, athletes of trampoline and tumbling were merged into one category, whereas acrobatics athletes also formed one group with those practicing aerobics. This was decided due to the similar nature of these sports, and in order to facilitate the analyses, as there were a relatively small number of athletes in each category initially.

Missing values for each parameter were noted and participants were excluded from the sample.

## 3. Results

### 3.1. Population Characteristics

A total of three hundred and nineteen (319) female gymnasts participated in the study. After controlling for a potential response bias via the SDS, 45 athletes with a score >9 were excluded from the study. Four athletes did not provide data regarding their height and body weight, and one participant did not complete the KIDMED questionnaire. Subsequently, these five were also excluded from all analyses.

Thus, a final number of 269 female gymnasts were included in the analyses for the present study. Characteristics of the participants are presented in Table 1.

### 3.2. Anthromometric Characteristics

The age, height, weight, and BMI of the participating athletes, according to their sport, are presented in Table 2. No differences were observed between the athletes of the different disciplines regarding their age (F(4,264) = 0.79, *p* = 0.533), however their weight (F(4,264) = 8.90, *p* ≤ 0.001), height (F(4,264) = 6.05, *p* ≤ 0.001), and BMI (F(4,264) = 10.71, *p* ≤ 0.001) were different between groups.

More specifically, post-hoc comparisons indicated that the athletes of artistic gymnastics exhibited a lower body weight when compared to the athletes practicing trampoline/tumbling (*p* = 0.007) and gymnastics for all (*p* ≤ 0.001). In parallel, the athletes practicing artistic gymnastics also had a lower stature when compared to their trampoline/tumbling counterparts (*p* = 0.012), those practicing acrobatics/aerobics (*p* ≤ 0.001), and gymnastics for all (*p* ≤ 0.001).

Moreover, rhythmic gymnastics athletes had lower body weight when compared to those practicing trampoline/tumbling (*p* ≤ 0.001), acrobatics/aerobics (*p* = 0.018), and gymnastics for all (*p* ≤ 0.001). Furthermore, they also exhibited the lowest BMI when compared to all other athletes (*p* = 0.012 for artistic gymnastics, *p* ≤ 0.001 for trampoline/tumbling, *p* = 0.014 for acrobatics/aerobics, and *p* ≤ 0.001 for gymnastics for all, respectively).

With regard to weight status, 84.0% of the athletes exhibited a normal BMI according to their age, with 10.4% (*n* = 28) being classified as underweight and the remaining 5.6% (*n* = 15) being overweight. No differences were observed between athletes of different disciplines (Χ^2^(8) = 30.49, *p* ≤ 0.001) regarding their weight status. The majority of underweight athletes were rhythmic gymnasts (*n* = 12), whereas, at the other end of the spectrum, the majority of overweight athletes were practicing gymnastics for all (*n* = 10).

### 3.3. Adherence to the MD

According to the KIDMED index, 34.9% of the athletes demonstrated a high degree of adherence to the MD, 56.1% had a score indicative of moderate adherence, and the remaining 8.9% followed a diet of low quality based on the MD principles. The level of adherence to the MD was different between weight status tiers. A significant difference was observed between KIDMED score, participant weight (F_(2,266)_ = 6.21, *p* = 0.002), and BMI (F_(2,266)_ = 4.78, *p* = 0.009) (Table 3). Post hoc analyses using the Bonferroni correction showed that athletes following an optimal diet had a significantly lower body weight when compared to those adhering to a diet of a low quality (difference −6.45 kg, 95%CI: −11.23 to −1.67, *p* = 0.004), and those with a moderate KIDMED score (mean difference −2.76 kg, 95%CI: −5.51 to −0.01, *p* = 0.048). With regards to the BMI, athletes following a diet of adequate quality had a significantly lower BMI when compared to those with a low quality diet (mean difference −1.44, 95%CI: −2.78 to −0.11, *p* = 0.028), and those following a diet of a moderate quality (mean difference −0.78, 95%CI: −1.55 to −0.02, *p* = 0.042).

Moreover, the KIDMED score was also correlated with the weight status category (Χ^2^(4) = 8.75, *p* = 0.035), while no significant associations were determined between KIDMED and the type of sport discipline (Χ^2^(8) = 7.35, *p* = 0.504), the competition level (Χ^2^(8) = 1.80, *p* = 0.988), or the training frequency (Χ2(4) = 8.85, *p* = 0.065). Finally, a negative correlation was noted between the KIDMED score and body weight (r = −0.258, *p* ≤ 0.001), as well as between the KIDMED score and BMI (r = −0.239, *p* ≤ 0.001).

In order to identify the ability of the KIDMED score to predict BMI, we performed linear regression analyses of the total sample and of different subgroups (Table 4). Firstly, in the total sample of the athletes, the linear regression demonstrated that each increase of one KIDMED unit corresponded to a decrease of 0.27 kg/m^2^ in the participants’ BMI (F(1,267) = 16.22, *p* ≤ 0.001, β = −0.27, 95%CI: −0.41 to −0.14) (Table 4—Model 1). As underweight is a serious and frequent problem in gymnastics, we also explored if this association meant that adherence to the MD was associated with a greater risk of being underweight. Firstly, all overweight athletes were excluded from the analyses in order to determine if the correlation remained. It was indicated that each increase of one KIDMED unit corresponded to a decrease of 0.19 kg/m^2^ in the participants’ BMI (F(1,252) = 10.26, *p* = 0.002, β = −0.19, 95%CI: −0.31 to −0.07) (Table 4—Model 2). Then, underweight athletes were excluded, and it was observed that each increase of one KIDMED unit corresponded to a decrease of 0.25 kg/m^2^ in the participants’ BMI (F(1,239) = 14.00, *p* ≤ 0.001, β = −0.25, 95%CI: −0.38 to −0.12) (Table 4—Model 3). Finally, the analysis was repeated using normoweight athletes only. It was revealed that each increase of one KIDMED unit corresponded to a decrease of 0.17 kg/m^2^ in the participants’ BMI (F(1,224) = 8.84, *p* = 0.003, β = −0.17, 95%CI: −0.29 to −0.06) (Table 4—Model 4).

### 3.4. Individual Eating Habits

**Figure 1:**
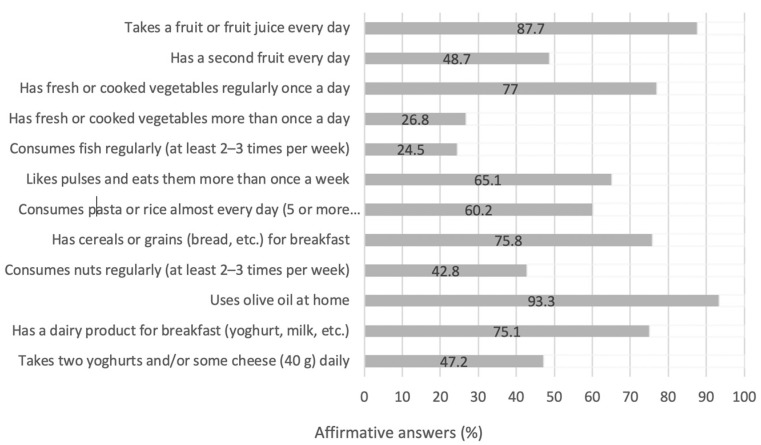
Percentages of participants adhering to healthy eating habits, according to the KIDMED.

The answers to individual KIDMED questions regarding positive eating habits are presented in Figure 1. A large percentage (87.7%) of female adolescent gymnasts consumed at least 1 portion of fruits or fruit juice on a daily basis, but less than half (48.7%) managed to consume a second portion every day. Accordingly, the majority (77.0%) consumed vegetables at least once per day, but a mere 26.8% consumed vegetables on a more frequent basis. 

The proposed consumption of fish, according to the MD (2–3 times/week), was only met by 24.5% of the gymnasts, and 65.1% reported eating pulses at least once weekly. Only 47.2% reported consuming two yoghurts and/or some cheese on a daily basis. With regard to pasta and rice, 60.2% reported a daily consumption. The majority of participants (93.3%) reported using olive oil at home and 42.8% reported a frequent consumption of nuts (>2–3 times/week).

Skipping breakfast regularly was reported among 27.5% of the sample, while 75.8% reported eating cereals or grains for breakfast, and 75.1% eating a dairy product for breakfast.

**Figure 2:**
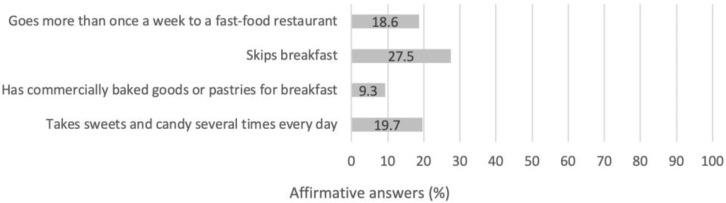
Percentage of participants following unhealthy eating habits, according to the KIDMED.

Moreover, 18.6% dined at a fast-food restaurant more than once-weekly, and 19.7% consumed sweets and candies several times each day, with 9.3% showing a preference towards commercially baked goods or pastries for breakfast (Figure 2).

The associations between participants’ eating habits and their BMI were evaluated using the independent samples *t*-test (Table 5). Differences were observed regarding the consumption of a second portion of fruit daily, the regular consumption of nuts, fast-food and breakfast skipping. Gymnasts who consumed a second fruit every day had a lower BMI than those who did not (*p* = 0.043). Accordingly, athletes meeting the proposed nuts consumption (>2–3 times/week) had a lower BMI than those that did not eat nuts as regularly (*p* *≤* 0.001). On the other hand, dining in a fast-food restaurant and breakfast skipping were both associated with a greater BMI (*p* ≤ 0.001 and *p* = 0.011, respectively).

Finally, a multiple linear regression model was used to estimate the relationship between BMI and the individual eating habits that were previously found to be significant. As detailed in Table 6, consuming nuts regularly (at least 2–3 times/week) and fast food dining at a frequency lower than once weekly significantly decreased BMI (F(4,264) = 7.23, *p* ≤ 0.001).

## 4. Discussion

The present study showed that a significant number of gymnasts do not adhere to the MD, while associations between total adherence to MD and specific eating habits and BMI were also present.

### 4.1. Anthropometric Characteristics

In the sample herein, the proportion of normoweight female adolescent gymnasts was 84.0%, with 10.4% of the sample being diagnosed as underweight. In further detail, the prevalence of underweight participants was 14% among girls practicing artistic gymnastics (14.0%) and double of that (28.6%) in the sample of rhythmic gymnasts. In addition, athletes belonging to these two disciplines (artistic and rhythmic gymnastics) demonstrated a lower body weight when compared to their counterparts from the other disciplines. Previous studies in adolescent female gymnasts indicate that those practicing artistic gymnastics have a smaller body size, lower body weight, and body fat percentage, especially when compared to their non-athlete peers [19,26,61,62,63]. Regarding rhythmic gymnasts, research has revealed that they exhibit low body fat levels [32,64,65], while their weight and BMI may be lower than required for their age, as a result of low energy availability [33].

A mere 5.6% of the participating athletes were classified as overweight, with the majority (2/3), practicing gymnastics for all. When compared to the prevalence of overweight among adolescent girls in the Greek community (10–29.5%), athletes herein exhibited a lower prevalence [50,66,67,68,69]. Among the examined gymnastics disciplines, the prevalence of overweight of athletes practicing gymnastics for all falls within the range of the Greek adolescent population’s prevalence of overweight. This result was expected, as this discipline in particular has much lower competitive requirements, with athletes not requiring a lower body weight to execute the exercises. Therefore, many girls with different body types and weights have the opportunity to participate in this discipline.

### 4.2. Adherence to the MD

Regarding diet quality, evaluated as the adherence to the MD, approximately one third of the participants herein exhibited optimal adherence to the MD. Approximately half of the girls followed a diet of a moderate quality, which was in need of improvement, and less that 10% exhibited a diet of a low quality. The present findings are in agreement with studies performed in Greek adolescents. The EYZIN study revealed a high degree of adherence to MD for 39.8% of the Greek adolescent population, with 9.7% following a diet of a low quality [70]. Similar results were also observed by Costarelli and associates [71], with 33.4% of their population exhibiting a high level of adherence to the MD. On the other hand, when compared to other studies performed in representative [72] and less representative samples of Greek adolescents [8,73], the results herein indicate a greater adherence to the MD. When compared to studies conducted in athletes, Zorzou [74] reported a lower degree of optimal adherence to the MD among Greek adolescent female athletes (18.4%), whereas Philippou [75] observed an optimal adherence to the MD among 21% of adolescent swimmers. On the other hand, a recent study in Spain reported that 41.63% of adolescent rhythmic gymnasts exhibited a high level of adherence to the MD [53].

The fact that nearly two-thirds of the adolescent athletes in our study do not fully adhere to the MD causes serious concern, as this means they may not consume enough nutrients, which are necessary for their health, growth, and athletic performance, thus being at risk of nutritional deficiencies. Previous research has demonstrated that adolescent gymnasts often have inadequate macronutrient and micronutrient intakes [32,34,36,37].

### 4.3. Adherence to the MD with Respect to Anthropometric Characteristics

In the present sample, correlations between adherence to the MD, body weight and BMI were observed. Athletes following the MD pattern to the optimal degree exhibited a lower body weight and BMI when compared to their counterparts with a low or moderate adherence to the MD. Linear regressions indicated that increases in the level of adherence to MD correlated with a lower BMI. This was present in the total sample, as well as in different subgroups according to weight status, i.e., in the group of normoweight athletes, in the group of underweight and normoweight athletes combined, and in the group of normoweight and overweight athletes combined. The relationship between a greater degree of adherence to the MD and achieving normoweight has been verified by previous research in the Greek adolescent population [8,48,50], as well as by a Spanish study on adolescent rhythmic gymnasts [53]. On the other hand, a recent study conducted in Spain using adolescent athletes from various sports failed to correlate an adherence to the MD and the anthropometric characteristics of participants (BMI, fat mass, muscle mass) [52].

An issue that arose, and was considered important for future consideration, was the negative association between KIDMED scores and BMI that was present when we excluded the overweight athletes. This result should be interpreted very carefully, as it may lead to the conclusion that better adherence to MD is linked to greater risk of being underweight, which is a serious concern for the sport of gymnastics. In our study, the mean BMI of the athletes was greater than the BMIs that were observed in other studies of adolescent gymnasts [29,53,64,76,77,78] and the percentage of underweight athletes was relatively small; this may affect the results of the analysis. Nevertheless, we believe that the association between MD adherence and BMI in non-normoweight athletes should be further examined in future studies. There may be the possibility that athletes that are underweight adhere to the MD, but at the same time they are not able to balance their energy intake. This may happen by eating less than the required amounts or by exercising more than their energy intake can cover (factors that we did not explore in our study), or even by using unhealthy weight control methods. On the other hand, in the group of normoweight athletes, which comprised the majority of our sample, the correlation between KIDMED and BMI was also present. This could mean that for the athletes who have a normal weight, adherence to MD may help them to control their weight and achieve a low, within-normal-limits weight that is considered beneficial for the execution of the exercises. Future research on this vulnerable population could clarify this issue.

### 4.4. Individual Eating Habits and BMI

Individual eating habits, as described in the distinct domains of the KIDMED questionnaire, also revealed associations between diet and BMI. In the sample of adolescent gymnasts, the majority (87.7%) of the participants reported consuming at least one portion of fruit or fruit juice daily, but less than half (48.7%) ate a second fruit every day. Consuming a second portion of fruit on a daily basis was correlated with a lower BMI. This finding has also been reported in other studies conducted among adolescents [79,80]. In comparison to the recent HBSC study conducted in Greek adolescents, a greater proportion of adolescent gymnasts appear to consume at least one fruit every day [6]. On the other hand, the German young Olympic Athletes’ Lifestyle and health management study (GOAL) reported that 61.9% of adolescent athletes consume fresh fruits on a daily basis [81].

Accordingly, a daily consumption of vegetables was reported by 77.0% of the participants herein, but only 26.8% ate vegetables more than once each day. The HBSC reported that 30–41% of the adolescent girls residing in Greece consume vegetables daily, but only 11.9% consume more than one portion [6,7]. Nevertheless, a large percentage of adolescent gymnasts in our study do not follow the national dietary guidelines for infants, children, and adolescents, which prescribes 2–3 fruit and 2–4 vegetable portions on a daily basis [82].

An interesting finding in the present study involved the correlation between the regular consumption of nuts and BMI. The participants who reported consuming nuts at least 2–3 times per week (42.8%) demonstrated a lower BMI when compared to the girls failing to consume nuts at the same frequency. This finding is in line with other studies conducted among adolescents. The International Study of Asthma and Allergies in Childhood (ISAAC) study, performed on participants from 35 countries, revealed that the adolescents consuming nuts three or more times per week exhibit a lower BMI when compared to those consuming nuts rarely, or never [83,84]. Another study found that adolescents who reported consuming nuts had a lower likelihood of being overweight or obese [85]. A possible explanation to this association may be that nuts provide significant amounts of essential fatty acids, protein and dietary fibers, thus promoting satiety [84]. Moreover, nut consumption has been correlated to improved overall diet quality in children and adolescents, by increasing their intake of essential nutrients [86].

The frequency of fast-food intake was also related to the BMI of participants. Those consuming fast-foods at least once weekly (18.6%) exhibited a greater BMI when compared to the rest of the sample. Many studies have reported similar findings. Systematic reviews indicate that a regular consumption of fast-food is related to a greater energy intake and a lower diet quality [87], whereas the consumption of ultra-processed foods during adolescence is associated with increased adiposity [88]. Fast-food consumption was also significantly correlated to the BMI of adolescents in the International Study of Asthma and Allergies in Childhood (ISAAC) study [56]. 

Finally, approximately one quarter of the female athletes herein reported skipping breakfast and, among those, a greater BMI was observed when compared to the athletes consuming breakfast regularly. In the HBSC study, a greater proportion of Greek adolescent girls reported skipping breakfast (34.6%) [7]. Similar findings have also been reported in studies conducted in adolescent and young adult populations [48,89,90,91,92]. Moreover, a number of studies have associated breakfast skipping with greater levels of body fat, insulin resistance, and metabolic syndrome in children and adolescents [80,93,94,95]. Finally, breakfast consumption has been associated with a higher daily micronutrients’ intake (especially B-vitamins, iron, calcium, magnesium, potassium, zinc, and iodine) than skipping breakfast [96].

### 4.5. Limitations

This study presents certain limitations that should be acknowledged. First, the cross-sectional design cannot address the causality of the results. Secondly, data collection was performed using self-reported questionnaires, such as the KIDMED questionnaire. Nevertheless, the KIDMED is a commonly used tool to determine adherence to MD and it is adapted to Greek population.

Self-reporting carries the risk of providing socially desirable answers. So, there is a chance that some athletes reported eating habits that do not actually describe their real habits. In order to limit this chance, we used the SDS to exclude the athletes that tended to provide answers that were perceived as “desired”, rather than true. Moreover, as gymnastics is a sport that is associated with a high prevalence of disordered eating, there is also a chance that some athletes might have undiagnosed typical or atypical eating disorders, or chose not to report relevant symptoms. This consists of a serious limitation, that we tried to overcome by asking the parents/guardians (through the consent form) if their child carried a nutrition-related diagnosis (such as diabetes, celiac disease, or anorexia nervosa). Moreover, the questionnaires were answered anonymously, in order to ensure that the athletes completed them as unaffected as possible.

Another significant limitation of the study is that anthropometric characteristics of the athletes (height and weight) were also self-reported, while there was no way to acquire additional data (such as body fat, muscle mass, waist circumference etc.). This methodology was adopted, as more detailed anthropometric measurements were not possible, due to COVID-19 pandemic measures. Therefore, it is considered possible that athletes may under- or over-report their weight or height. In order to limit this possibility, it was pointed out prior to the completion of the questionnaire that all data remained anonymous, and that it was important to provide accurate answers. Moreover, sexual maturation and body composition were not evaluated among the participants.

An additional limitation involves the sample size, which did not allow for the conduction of more complex regression models.

Finally, it should be highlighted that only female athletes were included in the study. This was a decision based on the nature of the sport of gymnastics, which attracts more female athletes than male athletes, and where female athletes are under greater pressure to maintain a low body weight.

### 4.6. Strengths of the Study

The present study is the first conducted in Greece, examining the level of adherence to the MD among adolescent female athletes of all gymnastics disciplines and all competition levels. Moreover, the included sample size is much bigger than that of previous studies conducted in the country among gymnastics athletes, and included female athletes of all types of disciplines and of all competition levels. Finally, female adolescent gymnasts from many different geographical areas of the country participated, providing a representative sample of the adolescent gymnast population.

## 5. Conclusions

A significant number of gymnasts do not appear to adhere to the MD principles. In parallel, female gymnasts who adhere to the MD tend to exhibit a lower body weight and BMI, although this finding should be further examined, especially regarding the athletes whose BMI is not within the normal ranges. The findings of the present study highlight the need for the development of targeted interventions that are aimed to improve the diet of adolescent athletes in Greece and other countries. These interventions should emphasize, among others, the role of the MD pattern regarding the achievement of a high quality diet, that could provide the nutrients that are essential for growth, wellness, and sport performance of adolescent athletes. Moreover, educational interventions should aim to provide information to athletes and coaches regarding the healthy ways to achieve an ideal body weight for the sport, and the adverse effects of using unhealthy and extreme dietary strategies.

Furthermore, we strongly suggest that each adolescent gymnast should be offered a personalized nutritional assessment and counseling frequently through the year. In this manner, underweight and overweight could be diagnosed timely, and athletes with weight-related problems would be guided to reach a normal and healthy weight, and control it through balanced nutrition.

Future research on adolescent athletes could further examine the possible association of specific eating patterns with BMI status or diet quality in different age groups, competition levels, and sport types, which should also include boys. Data regarding factors that were not addressed in our study, such as the influence of social media and peers, socio-economic status of the family, the beliefs and eating habits of parents, and even the beliefs and attitudes of the coaches, are also warranted, and should be included in future investigations.

## Figures and Tables

**Table 1:** Participants characteristics (*n* = 269).

Age (years)		13.89 ± 1.75
Body weight (kg)		47.44 ± 8.84
Height (cm)		157.4 ± 8.4
BMI (kg/m^2^)		19.00 ± 2.45
Weight status	Underweight/normoweight/overweight (*n*, %)	28 (10.4%)/226 (84.0%)/15 (5.6%)
Sport	Artistic gymnastics (*n*, %)	*n* = 50, 18.6%
	Rhythmic gymnastics (*n*, %)	*n* = 42, 15.6%
	Trampoline and tumbling (*n*, %)	*n* = 65, 24.2%
	Acrobatics and aerobics (*n*, %)	*n* = 22, 8.2%
	Gymnastics for all (*n*, %)	*n* = 90, 33.5%
Competition level: international/national/local (%)		20.2%/50.2%/29.6%
Duration of training: >8 years/6–8 years/<6 years (%)		27%/37.5%/35.5%
Weekly training frequency: ≥5 times/3–4 times/1–2 times		50%/34.3%/15.7%

BMI, body mass index.

**Table 2:** Anthropometric characteristics of female gymnastics athletes according to their sport (*n* = 269).

	Artistic Gymnastics (*n* = 50)	Rhythmic Gymnastics (*n* = 42)	Trampoline/ Tumbling (*n* = 65)	Acrobatics/ Aerobics (*n* = 22)	Gymnastics for All (*n* = 90)	Significance
Age (years)	13.69 ± 1.70	13.58 ± 1.64	13.99 ± 1.79	13.82 ± 1.56	14.08 ±1.84	*p* = 0.533
Body weight (kg)	43.9 ± 8.2	42.5 ± 6.8	49.3 ± 8.7 **^,†††^	49.5 ± 8.3 ^†^	49.9 ± 8.8 ***^,†††^	*p* ≤ 0.001
Height (cm)	152.8 ± 8.71	157.2 ± 8.8	157.8 ± 8.2 *	160.8 ± 7.1 ***	159.1 ± 7.6 ***	*p* ≤ 0.001
BMI (kg/m^2^)	18.65 ± 2.21 ^†^	17.07 ± 1.34	19.66 ± 2.18 ^†††^	19.02 ± 2.13 ^†^	19.62 ± 2.75 ^†††^	*p* ≤ 0.001
Underweight ^‡^ (*n*, %)	7 (14.0%)	12 (28.6%)	2 (3.1%)	1 (4.5%)	6 (6.7%)	*p* ≤ 0.001
Normoweight ^‡^ (*n*, %)	40 (80.0%)	30 (71.4%)	61 (93.8%)	21 (95.5%)	74 (82.2%)	
Overweight ^‡^ (*n*, %)	3 (6.0%)	0 (0.0%)	2 (3.1%)	0 (0.0%)	10 (11.1%)	

BMI, body mass index. ^‡^ classified according to the World Obesity criteria [59,60]. * statistically different when compared to the athletes from the artistic gymnastics group (* *p* ≤ 0.05, ** *p* ≤ 0.01, *** *p* ≤ 0.001); ^†^ statistically different when compared to the athletes from the rhythmic gymnastics group (^†^ *p* ≤ 0.05, ^†††^ *p* ≤ 0.001).

**Table 3:** KIDMED score categories (frequencies and percentages) by weight status, sport discipline, competition level and training frequency tiers.

		*n*	Low Adherence (KIDMED ≤ 3)	Moderate Adherence (KIDMED 4–7)	High Adherence (KIDMED ≥ 8)	Significance
Body weight (kg)		269	51.76 ± 7.56	48.07 ± 9.16	45.31 ± 8.11	*p* = 0.002 *
BMI (kg/m^2^)		269	19.88 ± 1.96	19.22 ± 2.71	18.43 ± 1.98	*p* = 0.009 *
Weight status	Underweight	28	0 (0.0%)	18 (64.3%)	10 (35.7%)	*p* = 0.035
	Normoweight	226	22 (9.7%)	121 (53.5%)	83 (36.7%)	
	Overweight	15	2 (13.3%)	12 (80.0%)	1 (6.7%)	
Sport discipline	Artistic gymnastics	50	4 (8.0%)	27 (54.0%)	19 (38.0%)	*p* = 0.504
	Rhythmic gymnastics	42	0 (0.0%)	26 (61.9%)	16 (38.1%)	
	Trampoline/tumbling	65	6 (9.2%)	40 (61.5%)	19 (29.2%)	
	Acrobatics/aerobics	22	3 (13.6%)	11 (50.0%)	8 (36.4%)	
	Gymnastics for all	90	11 (12.2%)	47 (52.2%)	32 (35.6%)	
Competition level	International	54	5 (9.3%)	29 (53.7%)	20 (37.0%)	*p* = 0.849
	National	134	10 (7.5%)	70 (59.0%)	45 (33.6%)	
	Regional	79	9 (11.4%)	42 (53.2%)	28 (35.4%)	
Weekly training frequency	≥5 times	134	7 (5.2%)	77 (57.5%)	50 (37.3%)	*p* = 0.065
	3–4 times	92	12 (13.0%)	55 (59.8%)	25 (27.2%)	
	1–2 times	42	5 (11.9%)	18 (42.9%)	19 (45.2%)	

BMI, body mass index; SD, standard deviation. * One way-analysis of variance.

**Table 4:** Linear regression analyses predicting BMI from KIDMED score among all adolescent female gymnasts and among different subgroups according to their weight status.

								95% CI	
		Constant	B	SE	β	*t*	*p*	Lower	Upper
Model 1	KIDMED (all athletes)	20.77	−0.27	0.07	−0.24	−4.03	<0.001	−0.41	−0.14
Model 2	KIDMED (exclude overweight)	19.97	−0.19	0.06	−0.2	−3.2	0.002	−0.31	−0.07
Model 3	KIDMED (exclude underweight)	20.94	−0.25	0.07	−0.24	−3.74	<0.001	−0.38	−0.12
Model 4	KIDMED (only normoweight)	20.15	−0.17	0.06	−0.19	−2.97	0.003	−0.29	−0.06

BMI, body mass index; CI, confidence intervals; SE, standard error.

**Table 5:** BMI values (kg/m^2^, mean ± SD) depending on the answers provided in the KIDMED questionnaire.

KIDMED Questions	Answers		Statistics	
	Yes	No	t	Significance
Takes a fruit or fruit juice every day	18.9 ± 2.5	19.6 ± 1.8	1.54	*p* = 0.124
Has a second fruit every day	18.7 ± 2.4	19.3 ± 2.5	2.03	*p***=** 0.043
Has fresh/cooked vegetables regularly once/day	19 ± 2.4	19.1 ± 2.5	0.35	*p* = 0.725
Has fresh/cooked vegetables ≥once/day	18.6 ± 2.3	19.2 ± 2.5	1.60	*p* = 0.110
Consumes fish regularly (≥2–3 times/week)	18.7 ± 2	19.1 ± 2.6	1.24	*p* = 0.213
Goes ≥once/week to a fast-food restaurant	20.1 ± 2.6	18.7 ± 2.4	3.67	*p* = 0.000
Likes pulses and eats them ≥once/week	18.8 ± 2.2	19.3 ± 2.8	1.60	*p* = 0.110
Consumes pasta/rice almost every day (≥5 times/week)	18.8 ± 2.3	19.3 ± 2.6	1.71	*p* = 0.088
Has cereals or grains (bread, etc.) for breakfast	19 ± 2.5	19 ± 2.4	−0.14	*p* = 0.884
Consumes nuts regularly (≥2–3 times/week)	18.4 ± 2.3	19.5 ± 2.5	3.65	*p* = 0.000
Uses olive oil at home	19 ± 2.4	18.6 ± 2.8	−0.70	*p* = 0.481
Skips breakfast	19.6 ± 2.7	18.8 ± 2.3	2.56	*p* = 0.011
Has a dairy product for breakfast (yoghurt, milk, etc.)	19 ± 2.5	18.9 ± 2.3	−0.42	*p* = 0.672
Has commercially baked goods or pastries for breakfast	18.8 ± 2.2	19 ± 2.5	−0.49	*p* = 0.621
Takes two yoghurts and/or some cheese (40 g) daily	19 ± 2.5	19 ± 2.5	−0.23	*p* = 0.816
Takes sweets and candy several times every day	19.2 ± 2.5	18.9 ± 2.4	0.80	*p* = 0.424

BMI, body mass index; SD, standard deviation.

**Table 6:** Multiple regression analysis predicting BMI among adolescent female gymnasts.

							95% CI	
Components of the Analysis	Answer	B	SE	β	*t*	*p*	Lower	Upper
Eating a second fruit every day	No	Reference category						
	Yes	−0.167	0.304	−0.034	−0.548	0.584	−0.765	0.432
Consuming nuts at least 2–3 times/week	No	Reference category						
	Yes	−0.83	0.305	−0.168	−2.723	0.007	−1.431	−0.23
Eating fast-food more than once a week	Yes	Reference category						
	No	−1.159	0.373	−0.184	−3.106	0.002	−1.893	−0.424
Skipping breakfast	Yes	Reference category						
	No	−0.625	−0.329	−0.114	−1.901	0.058	−1.272	0.022

CI, confidence intervals; SE, standard error.

## Data Availability

All data are available upon request to the first author.
